# HIV incidence and factors associated with testing positive for HIV among men who have sex with men and transgender women in Myanmar: data from community‐based HIV testing services

**DOI:** 10.1002/jia2.25454

**Published:** 2020-02-28

**Authors:** Vanessa Veronese, Michael Traeger, Zaw M Oo, Thet T Tun, Nwe N Oo, Htay Maung, Chad Hughes, Alisa Pedrana, Mark Stoové

**Affiliations:** ^1^ Disease Elimination Program Burnet Institute Melbourne Australia; ^2^ Department of Epidemiology and Preventive Medicine Monash University Melbourne Australia; ^3^ Burnet Institute Myanmar Yangon Myanmar; ^4^ Marie Stopes Yangon Myanmar; ^5^ Myanmar Business Coalition on AIDS Yangon Myanmar

**Keywords:** HIV prevention, men who have sex with men, transgender women, Myanmar, HIV epidemiology, HIV testing

## Abstract

**Introduction:**

In Myanmar, men who have sex with men (MSM) and transgender women (TW) are disproportionately affected by HIV, despite national HIV program scale‐up. However, limited HIV surveillance capacity prevents monitoring of epidemic trends and program impact. This study aimed to estimate HIV prevalence and incidence and explore associated sexual risk behaviours among MSM and TW clients attending HIV testing clinics in Myanmar.

**Methods:**

An electronic data management system was implemented in two community‐based, MSM and TW ‐tailored HIV testing clinics in Myanmar in August 2016. Unique client identifiers enabled prospective monitoring of service engagement, testing frequency and outcomes. We estimated HIV incidence and rate of HIV diagnosis at baseline testing visit among clients over a 15 month period. Correlates of HIV diagnoses were identified using multivariable logistic regression.

**Results:**

2794 MSM and TW were tested for HIV. At their baseline test, 38% of clients reported any previous testing and 93% reported being sexually active over the previous three months, with 74% reporting sex with casual male partners and 28% reporting consistent condom use with casual partners. 291 clients tested positive for HIV for the first time at baseline (10.4%; 95% CI: 9.3 to 11.6). Twelve incident cases were detected among 279 clients receiving ≥2 tests (incidence = 10.1 per 100 person‐years; 95% CI: 5.73 to 17.8). HIV diagnosis at baseline was significantly associated with being a transgender woman or a non‐openly disclosing man who has sex with men, age 26 to 39 years, and reporting no testing history.

**Conclusions:**

High HIV incidence and new diagnoses being associated with reporting no testing history points to undiagnosed HIV driving transmissions in Myanmar. Repeat testing was uncommon. HIV programs in Myanmar must focus on promoting frequent HIV testing alongside adequate coverage of education and primary prevention interventions among MSM and TW.

## 1 Introduction

Evidence of emergent HIV epidemics and prevalence estimates greater than 10% point to a disproportionate burden of HIV among men who have sex with men (MSM) and transgender women (TW) in Asia 1, 2, 3. This includes Myanmar, where estimated HIV prevalence among MSM and TW is 11% (results not disaggregated by sexual or gender identity), with prevalence estimates in the major cities of Yangon (27%) and Mandalay (22%) among the highest observed in the region 4, 5. In line with UNAIDS HIV elimination targets, a key part of Myanmar's national response to HIV is health systems improvement to advance progress towards 90‐90‐90 targets 5. However, sub‐optimal coverage and frequency of testing among key populations, including MSM and TW, remains a key challenge to HIV prevention in Myanmar and undermines a ‘test and treat' approach 5, 6.

Sexual minorities continue to experience stigma and discrimination in Myanmar. Homosexual sex remains criminalised under Myanmar's penal code, and while enforcement of this law appears rare, it acts to legitimise the intimidation or harassment of sexual minorities, contributing to stigma and discrimination as key barriers to HIV testing, treatment and care for MSM and TW 7, 8, 9. These experiences also contribute to many MSM and TW concealing their sexual orientation, particularly in health service contexts 8, 10. However, recent scale‐up of HIV programs, particularly decentralised HIV testing services run by local and international non‐government organisations (NGOs), has allowed for new models of community‐based and peer‐staffed HIV services. These services are now a common feature of Myanmar's HIV response and aim to promote greater engagement of key populations by circumventing known psycho‐social barriers to testing 10, 11, 12 in line with Myanmar's National Strategic Plan on HIV 5.

HIV epidemiological data in Myanmar is limited largely to infrequent integrated bio‐behavioural surveys 4, 13. While routinely collected HIV service‐level data can provide a platform for sustainable epidemiological data collection 14, there is currently no standard electronic data capture system within government clinics and no central integration of HIV testing and diagnosis data from NGO services. Limited recent estimates of HIV prevalence and an absence of data to monitor HIV incidence among MSM and TW hampers efforts to understand the impact of HIV programmatic responses in Myanmar.

In 2016, we developed and implemented an electronic data management system (eDMS) at two community‐based HIV testing clinics targeting MSM and TW in Yangon and Mandalay with the aim of strengthening data collection and reporting practices, and enabling the monitoring of HIV trends in a service delivery context. Using this data, we describe socio‐demographic characteristics, sexual behaviours and HIV testing behaviours and outcomes among clients; identify factors associated with HIV positivity, and; calculate HIV incidence among clients returning to test at the services.

## 2 Methods

### 2.1 Settings

The eDMS was implemented within two MSM and TW‐ focused community‐based drop‐in centres (DICs) in Yangon and Mandalay, the two largest urban areas in Myanmar with large, documented MSM and TW populations, relative to other areas 5. The DICs were managed by a local NGO Myanmar Business Coalition on AIDS (MBCA) and designed to attract MSM and TW in particular through the provision of discrete, non‐conspicuous HIV prevention services. Rapid point‐of‐care (RPOC) HIV testing was introduced into the DICs in March 2015, replacing the previous system of off‐site referrals. Health promotion and referred access to primary health care and STI services was freely available to clients, while incentives in the form of travel costs (2000 Myanmar Kyat, approximately equivalent to USD 2) were provided to promote uptake of HIV testing. HIV testing was conducted by trained nurses, and supported by trained MSM and TW peer educators who were responsible for pre‐ and post‐HIV test counselling.

### 2.2 Data collection

The eDMS was designed to replace previous paper‐based reporting systems used to record client visits and testing outcomes at the DICs and was piloted in Yangon and Mandalay over a 15‐month period (August 2016 to November 2017). At their first presentation to the DIC during this period, clients were registered into the system using a unique numeric identifier (UID) to enable prospective data linkage of testing episodes. Client contact information (name, residential township, phone number) and basic demographic details (age, male or female/transgender gender identity, highest level of education attained) were captured. Consistent with indigenous Myanmar labels of sexuality and gender expression among MSM and TW, clients were categorised through self‐identification as either: *apone* (men who typically do not disclose same‐sex sexual preferences or behaviour in most public spheres); *apwint* (individuals with male assignation at birth who openly identify as feminine, are sexually oriented towards men and otherwise regarded as TW); and, *thange* (behaviourally bisexual men who identify as heterosexual and often maintain relationships with women) 4, 8, 9, 10.

At first presentation and every subsequent visit during the 15 month pilot period, peer educators collected the following behavioural information during pre‐test counselling using handheld tablets: reason for HIV testing (condomless anal sex/injected drugs or shared needles/suspected HIV infection based on symptoms/routine testing/other); source of referral (self/referred by peer educator/referred by other organization); history of HIV testing (yes/no); time since last HIV test (<6 months/6 to 12 months/1 to 2 years/>2 years); and any contact with peer outreach worker in the past three months (yes/no). Clients who reported being sexually active in the past three months were asked about: sex with casual male partners (yes/no); sex with regular male partners (yes/no); sex with female partners (yes/no); condom use by partner type (never/occasionally/often/always); HIV status of regular male partner (HIV positive/HIV negative/unknown/prefer not to answer); experience of sexually transmissible infection (STI) symptoms in the past three months (urethral inflammation or discharge, dysuria, genital sores or ulcers, scrotal swelling or pain, inguinal or groin pain, genital warts, other symptoms; yes/no); paid money or gifts in exchange for sex (yes/no) or received money or gifts in exchange for sex in past three months (yes/no); and means of meeting casual male partners during past three months (multiple responses allowed: cruising sites/beauty salons/night clubs/online or phone/spa or massage venue/festival/other). A paper‐based version of all eDMS fields was developed in Myanmar language and available for use by DIC staff who did not wish to use the English‐version tablets, or when the tablets were unavailable. Information from these paper‐based forms was subsequently entered into the eDMS by a senior DIC staff member.

Following pre‐test counselling, a trained nurse authorised to conduct HIV testing used a two‐test algorithm – Alere Determine third generation HIV 1/2 (Abbott; Chicago, IL, USA), followed by HIV 1/2 STAT‐PAK Assay (Chembio; NY, USA) – to test clients for HIV. Final results were recorded as HIV positive/HIV negative/indeterminate in the eDMS, and delivered to the client with post‐test counselling. Three‐monthly testing was recommended to all HIV negative clients. Clients returning HIV positive or indeterminate results were referred to a local NGO for confirmatory HIV testing as part of initiation of free ART.

### 2.3 Consent

Service‐level permission was sought and granted from MBCA prior to the implementation of the eDMS to access to de‐identified client records. At the first visit during the pilot period, clients were informed of the use of their de‐identified data for research purposes and provided with a plain language participant informed consent form by peer educators. After describing the aims and duration of the research, permission was sought from clients to include their de‐identified clinic records in future data extractions during the pilot period; clients were informed of their ability to opt‐out and withdraw consent at any stage during this period. A specific field was built into the eDMS which, when checked, meant that records of clients who did not consent would be automatically excluded during data extraction.

### 2.4 Data management

All data entered into the eDMS between 01 August 2016 and 30 November 2017 were extracted for analysis. To maintain data continuity and fidelity during the eDMS pilot period and for ongoing reporting to program donors, DIC staff continued to use a data entry spreadsheet for recording HIV testing outcome, date of visit and basic demographic information at each testing episode. The eDMS and spreadsheet used the same UID, and the latter was used to validate testing episodes recorded in the eDMS and enhance data completeness. When consolidating the data sources, duplicate records matched by UID and occurring within 28 days of one another were consolidated into a single testing event; this cut off accounted for the reported low likelihood of repeat testing within one month and allowed for delays in data entry into either data source.

### 2.5 Analysis

Data collected during the first HIV test received at the DIC during the pilot period (‘baseline test') was used to describe socio‐demographic characteristics, reported HIV testing history and sexual behaviours, and HIV testing outcomes of MSM and TW. We describe the proportion of clients HIV positive at baseline; defined as the number of individual clients who tested positive at their first test, divided by the total number of individual clients with a valid test result during the pilot period. We examined factors associated with HIV positivity at baseline test through logistic regression and included all variables in both unadjusted and adjusted models. Collinear variables or variables with insufficient observations were excluded from the adjusted model.

Repeat testing behaviour was identified among clients receiving more than one HIV test at the service during the pilot period and median follow up time between first and last recorded test was calculated. HIV incidence was calculated among repeat testers, defined as the total number of incident cases divided by total follow‐up time accrued during the pilot period. Incident cases were defined as clients who returned a HIV positive test result during the pilot period following a negative result at their first valid (i.e. determinate positive or negative) test result. Follow‐up time was measured in person‐years (PY) and defined as the time between the first negative result and either a HIV positive result or last recorded negative result.

Analyses were conducted using Stata (Version 13, Stata Corp., College Station, TX, USA). Statistical significance in all analysis was set at *p* ≤ 0.05.

### 2.6 Ethics

Ethics approval was granted by the Alfred Hospital Ethics Committee, Australia and the Department of Medical Research, Myanmar.

## 3 Results

Between August 2016 and November 2017, 2801 individual clients received at least one test at Yangon or Mandalay DIC; seven of these clients returned an indeterminate test and were excluded from analysis. The remaining 2794 individuals were tested at least once at Mandalay (n = 1280; 45.8%) and Yangon (n = 1514; 54.2%). Among reporting clients, most identified as *apone* (44.7%) and 7.7% identified as *apwint* or TW. Most clients were aged 15 to 25 years (77.1%) and 63.5% were educated to a secondary or tertiary level. Approximately one third of clients reported a lifetime history of HIV testing (38.2%), with over half (55.4%) reporting a previous test at the DIC. Nearly all MSM and TW were sexually active during the past three months (93.0%). Among these clients, most reported sex with casual male partners (74.8%) and approximately one third reported sex with a regular male (31.9%), or a female partner (34.3%). Among those reporting sex with casual partners, 28.7% reported consistent use of condoms. The most commonly reported way of meeting casual male sex partners was via cruising sites (55.0%) and internet/phone‐based applications or websites (28.7%). Approximately one in five clients (18.9%) reported selling sex and 11.8% reported buying sex in exchange for money/gifts during the past three months. Ten per cent of clients reported experiencing STI symptoms in the past three months (Table 1).

**Table 1:** Socio‐demographic and sexual behaviours characteristics among clients at first valid test (n = 2794)

	n	%
Location		
Mandalay	1280	45.8
Yangon	1514	54.2
Gender		
*Apone*	1236	44.7
*Apwint*	214	7.7
*Thange*	1316	47.6
Age at registration		
15 to 25	931	77.1
26 to 39	237	19.6
40 to 60	39	3.2
Highest level of educational attainment		
Primary school or below (<5 years)	119	9.6
Middle school (6 to 9 years)	332	26.9
Secondary school (11 years)	480	38.9
Tertiary	304	24.6
Reported lifetime history of HIV testing		
No	746	61.8
Yes	461	38.2
Previous test conducted at DIC?		
No	205	44.6
Yes	255	55.4
Sexually active, past three months		
No	85	7.0
Yes	1123	93.0
Sex with female partner		
No	726	65.6
Yes	380	34.3
Sex with regular male partner		
No	762	68.3
Yes	352	31.9
Sex with casual male partner		
No	282	25.3
Yes	840	74.8
Consistent condom use with casual male partner during past three months		
No	589	71.3
Yes	237	28.7
Met casual sex partners through cruising sites		
No	544	45.0
Yes	666	55.0
Met casual sex partners using online/phone‐based apps or sites		
No	863	71.3
Yes	347	28.7
Sex sold past three months		
No	951	81.1
Yes	222	18.9
Sex bought past three months		
No	1040	88.2
Yes	139	11.8
STI symptoms past three months		
No	1063	89.7
Yes	123	10.3

*Apone, *non‐disclosing MSM*; Apwint,* transgender women*; Thange,* heterosexually‐identifying men who have sex with both men and women.

### 3.1 Baseline visit proportion HIV positive

Two hundred and ninety‐one clients tested positive at their first valid HIV test (proportion positive 10.4%; 95% CI: 9.3 to 11.6), of whom 206 were *apone* (70.8%), 41 were *apwint* (14.1%), 42 were *thange* (14.4%) and 2 were identity unknown (0.7%; data not reported). Unadjusted and adjusted correlates of testing HIV positive are presented in Table 2. In adjusted analysis, clients testing positive at baseline were significantly more likely to identify as *apone* (aOR 7.1; 95% CI: 3.0 to 16.5) or *apwint* (aOR 8.7; 95% CI: 3.1 to 24.9), be aged 26 to 39 years (aOR 2.8; 95% CI: 1.5 to 5.1), and were significantly less likely to report a lifetime history of HIV testing (aOR 0.3; 95% CI: 0.1 to 0.6) (Table 2).

**Table 2:** Associations between socio‐demographic and sexual behaviour characteristics and testing HIV positive at baseline test (n = 291)

	OR (95% CI)	aOR (95% CI)
Location		
Mandalay	1	1
Yangon	1.9 (1.5 to 2.5)*	0.8 (0.4 to 1.5)
Gender		
*Apone*	6.1 (4.3 to 8.5)*	7.1 (3.0 to 16.5)*
*Apwint*	7.2 (4.5 to 11.4)*	8.7 (3.1 to 24.9)*
*Thange*	1	1
Age at registration		
15 to 25	1	1
26 to 39	2.1 (1.3 to 3.2)*	2.8 (1.5 to 5.1)*
40 to 60	2.6 (1.1 to 6.1)**	0.5 (0.1 to 4.1)
Reported lifetime history of HIV testing		
No	1	1 (2.3 to 9.4)
Yes	0.7 (0.5 to 1.1)	0.3 (0.1 to 0.6)*
Previous test conducted at drop in centre?		
No	1	1
Yes	0.6 (0.3 to 1.1)	NA
Highest level of educational attainment		
Primary school or below (<5 years)	1	1
Middle school (6 to 9 years)	1.7 (0.7 to 4.2)	2.0 (0.6 to 6.7)
Secondary school (11 years)	1.6 (0.7 to 3.8)	1.7 (0.5 to 5.5)
Tertiary	3.2 (1.3 to 7.7)**	2.1 (0.6 to 7.1)
Sexually active, past three months		
No	1	1
Yes	1.0 (0.5 to 2.1)	NA
Sex with female partner		
No	1	1
Yes	0.2 (0.1 to 0.4)*	0.5 (0.2 to 1.4)
Sex with regular male partner		
No	1	1
Yes	2.8 (1.9 to 4.2)*	1.5 (0.8 to 2.9)
Sex with casual male partner		
No	1	1
Yes	0.7 (0.5 to 1.1)	NA
Consistent condom use with casual male partner during past three months		
No	1.6 (0.9 to 2.9)	1.6 (0.8 to 2.7)
Yes	1	1
Met casual sex partners through cruising sites		
No	1	1
Yes	0.9 (0.6 to 1.2)	1.4 (0.7 to 2.8)
Met casual sex partners using online/phone‐based apps or sites		
No	1	1
Yes	2.5 (1.7 to 3.6)*	1.5 (0.8 to 2.8)
Sex bought past three months		
No	1	1
Yes	1.5 (0.8 to 2.6)	2.4 (1.0 to 6.0)
Sex sold past three months		
No	1	1
Yes	1.0 (0.6 to 1.7)	0.8 (0.4 to 1.8)
STI symptoms past three months		
No	1	1
Yes	1.4 (0.8 to 2.4)	1.2 (0.5 to 2.6)

Incomplete or missing data means that denominators for variables may be less than the overall sample size. aOR, adjusted odds ratio; *Apone,* men who typically do not disclose same‐sex sexual preferences*; Apwint,* male gender assigned at birth who identify as feminine, and commonly regarded as transgender women*;* CI, confidence interval; NA, variables excluded from model due to collinearity; OR, odds ratio; *Thange,* heterosexual‐identifying men who have sex with both men and women.

* *p* < 0.001

** *p* < 0.01.

### 3.2 Repeat HIV testing and HIV incidence

The 291 clients who tested positive at their first test were excluded from incidence analysis. Among the remaining 2503 clients, 2224 had a single test during the pilot period (88.9%) and 279 were repeat testers (220 tested twice, 53 tested three times and six tested four times during the pilot period). There were no significant differences in sexual risk behaviours among repeat and single‐testing clients (data not reported). Among repeat testers, 12 incident HIV infections were detected across 344 post‐baseline visit testing events and 118.8 PY. Median follow‐up time was 147 days. All incident infections occurred among clients with male gender identity. HIV incidence was 10.1 per 100 PY (95% CI: 5.73 to 17.8). Limited number of incident infections and missing variable data prevented analysis of potential correlates.

## 4 Discussion

To our knowledge, this is the first study to report on correlates of HIV positivity and the first to generate an estimate of HIV incidence among MSM and TW attending a HIV testing service in Myanmar. Approximately 10% of MSM and TW tested positive at their baseline test, lower than the 14% HIV positivity among previously undiagnosed participants in the 2015 IBBS 4. We report on important factors associated with HIV positivity among MSM and TW not previously documented in Myanmar, including sexual identity, age, and HIV testing history. The rate of HIV incident infection among repeat testing clients was high relative to estimated incidence of HIV among MSM and TW reported elsewhere in the region 1, 15, 16. These findings, in combination with low levels of reported condom use among MSM and TW, carry important implications for HIV prevention activities in Myanmar, particularly in relation to enhancing uptake of regular HIV testing and timely access to treatment.

This study used service‐level data to estimate HIV burden and factors associated with HIV positivity at baseline among MSM and TW presenting for testing. As we elaborate below, service level data can be associated with substantial biases. However, with limited epidemiological data on MSM and TW in Myanmar and known challenges of achieving population‐based estimates for hard‐to‐reach and marginalised groups 17, the findings presented here are novel and support the utilisation of routine program data to increase understanding of HIV risk and burden among MSM and TW. Given the evidence of MSM and TW's ongoing predisposition to HIV risk 4, 8, our findings carry important implications for shaping future HIV prevention programming in Myanmar.

HIV incidence among MSM and TW documented in this study – 10.1 cases per 100 person years – is the first assessment of HIV incidence for any group in Myanmar, and demonstrates a higher rate of HIV transmission among MSM and TW compared to other estimates in Asia 1, 18, including among service‐engaged MSM in Bangkok 19. While HIV incidence was calculated among clients receiving more than one test during the pilot period and whose repeat testing behaviour may have been prompted by higher levels of risk than single testers, this finding points to a high level of HIV transmission among clients already engaged with a HIV prevention service. While the short follow‐up period and relatively small number of incident cases detected and missing variable data (discussed further below) precluded analysis of factors prospectively associated with HIV acquisition risk, other findings suggest that undiagnosed HIV in the context of infrequent testing and ongoing sexual risk behaviours may be a key contributor to ongoing transmissions 20, 21. While a lack of CD4 testing at HIV diagnosis in Myanmar prevents an assessment of time spent undiagnosed, the significant association between reporting no HIV testing history and baseline visit HIV positivity implies that some MSM and TW may have spent substantial time with unknown HIV.

Despite participating services recommending three‐monthly repeat testing, only about one in 10 clients repeat tested during the 15‐month pilot period. Moreover, clients who did not report a testing history at baseline were 70% less likely to repeat test during the pilot period (OR: 0.29; 95% CI: 0.2 to 0.4), with no significant difference in risk behaviours between those who did and did not repeat test (data not reported). While MSM and TW in Myanmar self‐report high levels of regular HIV testing 22, 23, the dissonance with data presented in this paper may suggest an overestimation of testing behaviours among MSM or TW, or ongoing barriers to enacting testing aspirations. Documented barriers to HIV testing among MSM and TW in Myanmar include experiences of stigma and discrimination, perceived unfriendliness of government or mainstream health staff, and avoidance of HIV prevention services manifestly catering for MSM and TW among those for whom concealment of sexual identity is a key priority 7, 8, 9, 10, 11, 12.

Encouraging frequent HIV testing and improving retention in HIV prevention programs must be a key focus of local HIV prevention strategies for MSM and TW in Myanmar 24. Novel testing models are needed to promote testing uptake and retention among MSM and TW. In particular, peer‐delivered testing and HIV self‐testing have demonstrated utility in increasing HIV testing uptake among stigmatised populations in other Asian settings 25, 26. Both models are backed by a documented willingness among MSM and TW to access such services 11, 27 and a supportive policy environment that is conducive to the introduction of novel testing modalities in Myanmar 5. These factors provide an opportunity to diversify testing delivery approaches as a key strategy to enhance regular HIV testing, and should be considered alongside condom reinforcement programs and consideration of HIV pre‐exposure prophylaxis (PrEP) (undertaking PrEP demonstration projects is currently recommended in the national strategic plan but yet to occur) 5, 22.

Sexual identity was significantly correlated with baseline HIV positivity. Transgender identity was identified as a significant predictor of prevalent HIV; *apwint* clients were more than eight times more likely to test positive at baseline than *thange* or behaviourally bisexual men. A range of documented risk factors may contribute to the disproportionate HIV burden among TW relative to other sexual and gender identities in Myanmar, including being more likely to report receptive anal sex positioning, higher numbers of sexual partners, lower levels of condom use, earlier sexual debut, and experiences of forced sex 5, 7, 28, 29. Globally, TW are nearly 50 times more likely to have HIV than all adults of reproductive age 30, while in Asia, TW are also less likely to be retained in ART care 31, suggestive of broad structural and social barriers for TW across the HIV care continuum. Additionally, *apone* clients – men who are sexually oriented towards other men but typically do not openly identify as gay – had seven‐times higher risk of HIV positivity at baseline than *thange*. Across Asia, non‐disclosure of sexual identity has been associated with higher levels of condomless sex 32 and a lower uptake of testing for HIV 33, 34 and STIs 35, which may explain some of the increased vulnerability to HIV observed among *apone* clients in this study. Awareness of sexual orientation is an important precursor to provider‐initiated HIV testing 36, and non‐disclosure of sexual behaviours may hinder the provision of relevant and tailored sexual health information, including assessments of eligibility for biomedical HIV prevention options. Our findings corroborate reports of differential levels of HIV vulnerability and access to HIV services among sexual identities in Myanmar 4, 11, 23, 29 and support the need for HIV prevention strategies tailored to the specific needs of MSM and TW sub ‐populations.

Our findings should be considered with the following limitations in mind. First, as mentioned above, the self‐selection bias inherent in service‐level data may limit the generalizability of these findings to the wider MSM and TW population in Myanmar. Engagement in HIV prevention and testing may be motivated by perceptions of HIV risk which may overestimate our estimates of HIV burden. Conversely, the risk of HIV may be lessened among clients actively engaging in HIV prevention services. Second, our incidence estimates may be underestimated due to the limited number of clients engaging in repeat testing behaviour – potentially an artefact of the short period of observation. Third, this data was collected from MSM and TW residing in the two largest cities in Myanmar with the highest prevalence of HIV 5, which are also well serviced by local and international NGOs. MSM and TW in this study may therefore have relatively high levels of access to HIV prevention and testing services and our findings may not be generalizable to MSM and TW who are not engaged with services or reside in peri‐urban or rural areas of Myanmar. Finally, we acknowledge that implementation issues encountered during the piloting of this new electronic system resulted in some missing socio‐demographic and sexual behaviour data which limited our ability to further characterise incidence infections and the potential representativeness of our findings. This includes missing data on gender identity and the relatively small number of participants self‐identifying as *apwint* which prevented a reliable calculation HIV prevalence and incidence by gender identity. Nonetheless, we believe that given the absence of data to date on HIV epidemiology among MSM and TW in Myanmar, the novelty of the data collection system in the local context and the implicit need for more robust data collection systems to better monitor HIV infection and to inform programming priorities in Myanmar, the findings presented in this paper represent an important starting point for further research into factors associated with HIV infection among MSM and TW in Myanmar.

## 5 Conclusions

Despite the implementation challenges noted above, the use of electronic health data in low‐income countries remains limited and the data presented here highlights both the utility and potential of novel electronic data collection system to capture data that can inform HIV prevention priorities. This pilot project demonstrated high HIV incidence among MSM and TW clients, relative to estimates from the region, combined with high levels of risk behaviours and infrequent engagement with HIV testing services. These factors strongly support the scale‐up of HIV prevention activities, the prioritisation of regular HIV testing and enhanced retention among MSM and TW in Myanmar, as well as consideration of more contemporary testing and prevention approaches such as peer‐delivered and self‐HIV testing, and PrEP into the national HIV response in Myanmar. Such strategies must be cognisant of socio‐demographic and other factors that may influence engagement with HIV testing services. This study supports the utilisation of community‐based HIV testing services as an important source of ongoing epidemiological data in Myanmar, as well as in other low‐resource settings in the region.

## Competing interests

None to declare.

## Authors' contributions

VV, MS and CH designed the study and developed the data collection tools. VV, ZMO, TTT, NNO and HM managed in‐country data collection efforts. Data cleaning and analysis was led by VV, MT and MS. AP provided input into data analysis and manuscript preparation. All authors have read and approved the final manuscript.
